# Development and validation of a weather-based warning system to advise fungicide applications to control dollar spot on turfgrass

**DOI:** 10.1371/journal.pone.0194216

**Published:** 2018-03-09

**Authors:** D. L. Smith, J. P. Kerns, N. R. Walker, A. F. Payne, B. Horvath, J. C. Inguagiato, J. E. Kaminski, M. Tomaso-Peterson, P. L. Koch

**Affiliations:** 1 Department of Plant Pathology, University of Wisconsin-Madison, Madison, Wisconsin, United States of America; 2 Department of Plant Pathology and Entomology, North Carolina State University, Raleigh, North Carolina, United States of America; 3 Department of Entomology and Plant Pathology, Oklahoma State University, Stillwater, Oklahoma, United States of America; 4 Department of Plant Sciences, University of Tennessee, Knoxville, Tennessee, United States of America; 5 Department of Plant Science and Landscape Architecture, University of Connecticut, Storrs, Connecticut, United States of America; 6 Department of Plant Science, Pennsylvania State University, State College, Pennsylvania, United States of America; 7 Department of Biochemistry, Molecular Biology, Entomology, and Plant Pathology, Mississippi State University, Starkville, Mississippi, United States of America; Universita degli Studi di Pisa, ITALY

## Abstract

Dollar spot is one of the most common diseases of golf course turfgrass and numerous fungicide applications are often required to provide adequate control. Weather-based disease warning systems have been developed to more accurately time fungicide applications; however, they tend to be ineffective and are not currently in widespread use. The primary objective of this research was to develop a new weather-based disease warning system to more accurately advise fungicide applications to control dollar spot activity across a broad geographic and climactic range. The new dollar spot warning system was developed from data collected at field sites in Madison, WI and Stillwater, OK in 2008 and warning system validation sites were established in Madison, WI, Stillwater, OK, Knoxville, TN, State College, PA, Starkville, MS, and Storrs, CT between 2011 and 2016. A meta-analysis of all site-years was conducted and the most effective warning system for dollar spot development consisted of a five-day moving average of relative humidity and average daily temperature. Using this model the highest effective probability that provided dollar spot control similar to that of a calendar-based program across the numerous sites and years was 20%. Additional analysis found that the 20% spray threshold provided comparable control to the calendar-based program while reducing fungicide usage by up to 30%, though further refinement may be needed as practitioners implement this warning system in a range of environments not tested here. The weather-based dollar spot warning system presented here will likely become an important tool for implementing precision disease management strategies for future turfgrass managers, especially as financial and regulatory pressures increase the need to reduce pesticide usage on golf course turfgrass.

## Introduction

Golf course turfgrass maintenance is an economically important component of the U.S. horticulture industry, comprising a significant portion of the $70 billion contribution the golf industry made to the United States economy in 2011 [1]. Arguably the single most significant disease of golf course turfgrass in temperate climates is dollar spot, caused by the fungal ascomycete *Sclerotinia homoeocarpa* F. T. Benn [2–4]. Dollar spot is a foliar disease that occurs on most types of both cool- (C3 photosynthesis) and warm- (C4 photosynthesis) season turfgrasses. Species affected include bentgrasses (*Agrostis sp*.), bermudagrass (*Cynodon sp*.), bluegrass (*Poa sp*.), buffalograss (*Buchloë dactyloides*), fescue (*Festuca sp*.), ryegrass (*Lolium sp*.), seashore paspalum (*Paspalum vaginatum*), and zoysiagrass (*Zoysia sp*.). On golf course surfaces that are mowed between 2 and 12 mm, infection results in numerous circular infection centers of tan or brown turf that measure 2 to 5 cm in diameter, and if not controlled, dollar spot can result in sunken areas that affect ball roll, contribute to weed and algae encroachment, and lead to plant death [3–4].

FT Bennett first identified *S*. *homoeocarpa* as the causal agent of dollar spot in 1937, yet a clear understanding of its biology and epidemiology remain elusive today [5]. The broad range of optimal infection temperatures (15 to 30°C) and a relative lack of effective cultural control techniques have resulted in heavy reliance on chemical fungicide applications for achieving acceptable disease control [3, 4, 6]. This heavy reliance on chemical control methods costs the average golf facility more money to control than any other turfgrass disease, has led to widespread fungicide resistance within *S*. *homoeocarpa* populations, and raises numerous environmental and human health concerns [4, 6–9].

Timing fungicide applications in turfgrass and other agronomic and horticultural crops has traditionally relied on strict calendar-based methods that result in reapplication intervals ranging from 7 to 28 days [4]. To time fungicide applications more appropriately to prevent disease outbreaks, weather-dependent predictive algorithms have been developed using risk indices or linear regression techniques to predict infection periods in numerous agricultural and horticultural crops. Some of these predictive models have been used in advisory systems for food crops such as peanuts [10], spinach [11], and carrot [12]. In turfgrass, regression-based advisories have been developed for brown patch (caused by *Rhizoctonia solani*) of perennial ryegrass [13] and dead spot (caused by *Ophiosphaerella agrostis*) of creeping bentgrass (*Agrostis stolonifera* L.) [14]. Fidanza et al. used mean relative humidity and minimum air temperature in a second order linear regression to predict the environmental favorability for brown patch outbreaks [13]. The model accurately predicted all major brown patch outbreaks over a 3-year span and was able to provide comparable brown patch control compared to a calendar-based application schedule with 29% fewer fungicide applications. This research demonstrates that combining detailed weather data with a regression-based prediction model can improve the ability to predict turfgrass disease epidemics and time fungicide applications more appropriately.

Previous attempts at predicting dollar spot epidemics on turfgrass using risk index-based algorithms have been made by Hall [15] and Mills and Rothwell [16]. More recently, Ryan et al. developed a growing degree-day (GDD) model to predict the initial onset of dollar spot symptom development on six creeping bentgrass cultivars with varying susceptibility to dollar spot development [17]. Hall determined that 48 consecutive ‘wet’ hours with an average daily temperature of at least 22°C was required for dollar spot epidemics to occur on creeping bentgrass [15]. Alternatively, if temperatures were below 22°C, three or more consecutive ‘wet days’ were required to initiate the epidemic. Mills and Rothwell recommended a fungicide application when maximum air temperature was ≥25°C and maximum relative humidity was ≥90% during any 3 days of a 7-day period [16]. However, a two-year study comparing the Mills-Rothwell and Hall models demonstrated that the Mills-Rothwell model tended to over predict the number of infection periods during a growing season while the Hall model under-predicted infection periods and resulted in widespread disease development relative to a calendar-based approach [18]. The Ryan et al. GDD model is accurate in predicting initial dollar spot onset in certain creeping bentgrass cultivars, but makes no attempt to predict dollar spot development the remainder of the growing season. The reason for the inability of these models to accurately predict dollar spot development throughout the growing season is not entirely clear but likely includes a lack of precision and accuracy of weather monitoring instruments and/or data inputs, incorrect thresholds for the weather variables chosen, under emphasis of leaf wetness parameters, or the lack of use of other biologically important weather variables [4].

Logistic regression is a promising alternative to traditional linear regression techniques for the development of more effective disease warning systems. Logistic regression allows the user to develop a model that predicts the likelihood that an event (ex. disease event) is going to occur based on binary data (1 = event occurrence, 0 = no event occurrence). To date logistic regression has not been widely used in turfgrass pathology, but has been tested extensively and proven effective in other plant pathogen systems [19–21]. The advantages of this technique in developing a plant disease prediction model include the ability to use simple binary data rather than more complicated approaches such as spore sampling or counting lesions, etc. It also facilitates the development of a model that can deliver the likelihood that an epidemic is going to occur, similar to a daily weather report. This probability of epidemic output allows the end user to establish his/her own action threshold based on their experience and location rather than using one specific threshold. In this way, the logistic regression approach is extremely versatile and has great potential in the development of a novel dollar spot warning system.

Determining the efficacy of a disease-predictive model across numerous sites and years is a critical part of developing an effective plant disease warning system. Though temporal and spatial validation requires significant time and money, it is paramount to ensure reliable prediction over a broad range of conditions before public deployment. Multi-site and multi-year datasets can often be complex, and meta-analysis can be used to test hypotheses using these kinds of datasets [22–23]. Meta-analysis was originally developed for analysis in the social sciences and medical sciences and recently has been adopted in plant pathology to examine results from many studies in a single, yet statistically powerful, analysis [22]. For example, meta-analysis was recently used in strawberry production to test the utility of using a disease warning system for advising fungicide applications versus using a calendar-based approach in applying fungicide for various fruit rots [24]. Similar techniques could be useful in calculating the probability of success in using a disease warning system over many site-years in turfgrass.

The objectives of this paper were to (1) use logistic regression to develop a novel dollar spot warning system for use on turfgrass and (2) to validate the effectiveness of the dollar spot warning system for advising fungicide applications over a wide range of geographic sites over multiple years (site-years).

## Materials and methods

### Logistic model development field studies

Research plots were established at the Oklahoma State University Plant Pathology Research Farm located in Stillwater, OK in 2008 and 2009; and at the University of Wisconsin O.J. Noer Turfgrass Research Center located in Madison, WI and the University Ridge Golf Course located in Verona, WI during the 2008, 2009, and 2010 growing seasons (Table 1). Research plots were established on mature creeping bentgrass (*Agrostis stolonifera*) mowed at 2.5 mm at both the Oklahoma and Wisconsin research stations and mowed at 12.7 mm at University Ridge Golf Course.

**Table 1:** Year, state, location, site, season, and number of observations when dollar spot severity data were collected for the development of logistic regression models.

Year	State	Location^a^	Site^b^	Season^c^	Observations (n)
2008	Oklahoma	OSU	GH	Spring	1,044
	Oklahoma	OSU	GH	Fall	630
2009	Oklahoma	OSU	GH	Spring	756
	Wisconsin	OJN	GH	Fall	36
	Wisconsin	OJN	GH	Summer	1,134
	Wisconsin	UR7	FH	Fall	36
	Wisconsin	UR7	FH	Summer	1,134
	Wisconsin	UR14	FH	Fall	36
	Wisconsin	UR14	FH	Summer	1,134
2010	Wisconsin	OJN	GH	Summer	414
	Wisconsin	UR7	FH	Summer	414
	Wisconsin	UR14	FH	Summer	414
				TOTAL	7,182

^a^ OSU = Oklahoma State University, Turfgrass Research Center, Stillwater, OK; OJN = O.J. Noer, Turfgrass Research Center at the University of Wisconsin-Madison; UR7 = fairway #7 at University Ridge Golf Course, Verona, WI; UR14 = fairway #14 at University Ridge Golf Course, Verona, WI

^b^ GH = greens height of cut for creeping bentgrass, FH = fairway height of cut for creeping bentgrass

^c^ Season when epidemic initiated

The creeping bentgrass cultivar ‘Penncross’ was used at the WI plots and ‘SR1020’ was used in OK and both are highly susceptible to *S*. *homoeocarpa* infection. Plots were arranged in a randomized complete block design (RCBD) with six replications (blocks) with three treatments. Individual plots were 0.91 m x 0.91 m and the entire experimental area at each location was 2.73 m x 5.46 m. Plots were either treated with a fungicide or not treated to determine the contribution of fungicide effects on model parameters. Treatments included a non-treated control, a curative fungicide treatment applied within 48 hours of dollar spot symptom appearance, and a calendar-based fungicide treatment applied every two weeks. Vinclozolin (Curalan 50EG; BASF Corporation, Research Triangle Park, NC) at 1.36 kg a.i. ha^-1^ was used for both the preventative and curative treatments in Oklahoma. At both Wisconsin sites a tank mixture of propiconazole (Banner Maxx 1.3EC; Syngenta Professional Products Greensboro, NC) at 0.50 kg a.i. ha^-1^ and chlorothalonil (Daconil WeatherStik 6F; Syngenta Professional Products Greensboro, NC) at 5.72 kg a.i. ha^-1^ was applied for the calendar-based treatments, while a tank mixture of propiconazole at 1.00 kg a.i. ha^-1^ and chlorothalonil at 11.44 kg a.i. ha^-1^ was applied for the curative treatments. Treatments were applied using a CO_2_—pressurized boom sprayer at 276 kPa equipped with two XR Teejet 8004VS (8008VS in OK) nozzles. All fungicides were agitated by hand shaking and were applied in the equivalent of 814 L of water ha^-1^. Dollar spot symptom development was assessed daily by counting the number of new infection centers present that day. New infection centers were then painted with spray paint so that infection centers were not counted more than once during the evaluation period. This technique was used to reduce the likelihood of collinearity during model development and ensure that only new infections centers were counted each day. Infection centers were counted throughout an entire growing season from May until September in each year. If no new infection centers were present on a given day then zeros were used to designate daily symptom development.

### Weather data collection

Weather data were collected at each research site using Watchdog 2700 weather stations (Spectrum Technologies, Aurora, IL). Onboard sensors measured air temperature, dew point, relative humidity, rainfall, and wind speed/direction. Additional sensors were added as accessories from Spectrum Technologies and included a leaf wetness sensor (Item# 3666), Watermark soil moisture sensor (Item #6450WD), and external soil temperature sensor (Item # 3667). Stations were mounted to a solid post at a height of 1.82 m over the turf canopy immediately adjacent to each research site. The soil moisture sensor and soil temperature sensor were placed at a depth of 10-cm depth. The leaf wetness sensor was painted with a single coat of white latex paint and affixed pointing north at a height of 30.5 cm over the turfgrass canopy. Data loggers from Spectrum Technologies were programmed to record data every minute and average data points for each hour. Hourly weather data were used for subsequent analysis.

### Dollar spot warning system development

Weather data were converted to five-day moving averages for each variable of interest. Initial testing of moving average duration suggested that a five-day average was most correlated with disease development (*data not shown*). Five-day moving averages have been used in other models developed to predict diseases caused by *Sclerotinia* species [10, 25]. A binary dummy variable for fungicide use was also developed where a 1 indicated the application of fungicide (preventatively or curatively) and 0 indicated no fungicide was applied. Additionally, disease data were converted to a binary form where 1 indicated new dollar spot development for each day and 0 indicated no new dollar spot development. Data were then averaged across replicates at each site within each date (e.g. a single mean across replicates was generated), and any infection center average greater than 0 was converted to a score of 1. The plot areas were small and variability was low among replicates; therefore, replicates were used to simply increase precision in observations. Logistic regression was used to generate disease prediction models because the dependent and some independent variables were dichotomous with a binomial distribution of errors. Logistic regression is used to estimate the probability of an event occurring, unlike typical least-squares regression where the intent is to estimate intensity of the event. Correlation analysis (Kendall’s Tau) was conducted immediately prior to model development using logistic regression techniques to determine the non-parametric level of association of independent variables with the dependent variable using PROC CORR Kendall (SAS Institute, Cary, NC). Variables with a correlation coefficient of ≥ 0.1, relative to the dependent variable, were included in the model building procedures.

Model building was performed using PROC LOGISTIC (SAS Institute, Cary, NC). All variables highly correlated with disease were added to the model sequentially using the STEPWISE option. A test for multi-collinearity was performed as described by Allison [26]. The tolerance and variance inflation of the variables were calculated using the PROC REG procedure adjusted by the weight matrix. Tolerances below 0.40 were considered as the cutoff for contributing to variance inflation and variables were removed from the model if they fell below this level. Model goodness-of-fit was evaluated using the LACKFIT option to calculate the Hosmer and Lemeshow Goodness-of-fit test. This option allows for testing the null hypothesis that the resulting model IS a good fit. A non-significant χ^2^ would indicate that the null hypothesis is not rejected and the model is a good fit [26]. Deviance residuals were also calculated and plotted against estimated probabilities to check for any data points out of range among the rest of the data set [26]. The receiver operator curve (ROC) was also evaluated for all models to check specificity and sensitivity. Specifically, the area under the ROC was calculated from the c-statistic in the output table in PROC LOGISTIC. Models with higher values of c were considered a better fit of the data, with acceptable discrimination above 0.7 [27].

The resulting models can be written as follows:

$$Logit(\mu )=\beta_{o}+\beta_{1}X_{1}+\beta_{2}X_{2}+\cdots +\beta_{p}X_{p}$$ (1)

where X_*p*_ are predictor variables and β_*p*_ are parameter estimates. The logistic models describing the estimated probability of an event can be written as follows:

$$\pi (\mu )=\frac{e^{logit(\mu )}}{{1+e}^{logit(\mu )}}$$ (2)

where π (μ) is the estimated probability of infection center development, which is the logit transformation.

### Validation of the dollar spot warning system in the field

Additional research sites were established between 2011 and 2016 to validate and refine specific model action thresholds (probability cutoff) to advise an application of fungicide (Table 2). Model validation sites were established at the O.J. Noer Turfgrass Research Facility (Madison, WI), University Ridge Golf Course (Verona, WI), Oklahoma State University Turfgrass Research Facility (Stillwater, OK), the Joseph E. Valentine Turfgrass Research Center (State College, PA), the West Tennessee Turfgrass Research and Education Center (Knoxville, TN), the Plant Science Research and Education Facility (Storrs, CT), and the R. R. Foil Plant Science Research Center (Starkville, MS). All validation plots were conducted on creeping bentgrass maintained at 12.7 mm except for the plots at University Ridge and R. R. Foil, which were conducted on creeping bentgrass at 2.5 mm and 3.2 mm, respectively. Each site included three treatments: a non-treated control that did not receive any fungicide, a standard calendar-based fungicide program made on a 21-day reapplication interval, and a fungicide program where application timing was based on the dollar spot model predictions. The fungicide applied in both programs was Banner MAXX at the rate of 1.00 kg a.i. ha^-^1. Based on preliminary data from the model development, a model probability output of 20% was used in most of the validation studies as the initial spray threshold (data not shown). Plots were again 0.91 m x 0.91 m in size and arranged in a RCBD with six replicates. Fungicide application and data collection were performed in the same manner as the model development studies.

**Table 2:** Research sites used in model validation and threshold refinement for deployment of dollar spot logistic regression models for use in advising fungicide applications. All sites were included in the meta-analysis comparing the efficacy of the logistic regression model to a calendar-based spray program. All sites were tested on creeping bentgrass.

State^z^	Location	Years
WI	Madison (OJ Noer)	2010–2011
WI	Madison (Univ Ridge GC)	2010–2011
OK	Stillwater	2010–2011
PA	State College	2011–2012
TN	Knoxville	2011–2012
MS	Starkville	2011–2012
WI	Madison (OJ Noer)	2014–2016
WI	Madison (Univ Ridge GC)	2014–2016
WI	Madison (OJ Noer)	2014–2016
WI	Madison (Univ Ridge GC)	2014–2016
CT	Storrs	2015–2016

^z^ CT = Connecticut, MS = Mississippi, OK = Oklahoma, PA = Pennsylvania, TN = Tennessee, WI = Wisconsin

Two additional sets of studies (threshold testing studies and a reduced-impact disease management study) were conducted at the O.J. Noer Turfgrass Research Facility and University Ridge Golf Course from 2014 through 2016. The threshold testing study consisted of eight treatments arranged in a RCBD with four replicates and a plot size of 0.91 m x 3.0 m. The treatments were a non-treated control, fungicide applied on a 21-day reapplication interval, and fungicide applied at model action probabilities of 10%, 20%, 30%, 40%, 50%, and 75%. In 2015 and 2016, the model probabilities tested were narrowed to 10%, 15%, 20%, 25%, and 30%. The fungicide applied in each treatment was Banner MAXX at the rate of 1.00 kg a.i. ha^-1^. The data from the reduced-impact study were part of a larger study and only the dollar spot data related to the usage of the warning system are published here. The study consisted of four treatments arranged in a RCBD with four replicates and a plot size of 1.8 m x 3.0 m. The treatments were a non-treated control, a fungicide program applied using calendar-based methods, a fungicide program using the dollar spot warning system to time applications, and a program using the dollar spot warning system to time fungicide applications but only using products that were assumed to have a lower environmental impact level as assessed by the Environmental Impact Quotient [28]. Fungicide application and data collection were performed in the same manner as the aforementioned studies. Dollar spot results from the 2015 and 2016 threshold refinement study were converted to area under the disease progress curve (AUDPC) using the trapezoidal method and then divided by the number of days of each study [29]. Data were subjected to an analysis of variance using PROC MIXED (Version 9.4; SAS Institute, Cary, NC) and means were separated by Fishers Least Significant Difference using the PDMIX macro in SAS [30].

A meta-analysis approach was used to validate the accuracy of specific models across an array of environments and to refine action thresholds for advising fungicide applications. Madden and Paul [22] and Paul et al. [31] describe the techniques for meta-analysis in detail. Briefly, a primary mixed-model analysis of variance (ANOVA) was performed using PROC GLIMMIX (Version 9.4; SAS Institute, Cary, NC) for each trial within each year (study or K). Replicate was treated as a random effect and fungicide program (product nested within application decision method and action threshold) as a fixed effect in a model describing dollar spot control as a percentage relative to the non-treated control. Dollar spot control (C) was calculated as follows:

$$C=100-(\frac{F_{program}}{F_{Non-treated}}X\;100)$$ (3)

where F_program_ is the number of dollar spot foci in the treatments where a fungicide application program was used and F_non-treated_ is the number of foci in the non-treated plots. Dollar spot foci were assessed every one to two weeks by counting total number of infection centers in an entire plot (experimental unit). Unlike in the model development studies, foci were not painted, thus foci may have been counted more than once on subsequent assessments. The overall mean control effect size ($\bar{C}$) was then calculated for each study from the ANOVA as follows:

$$(\bar{C})=C_{program}-C_{calendar}$$ (4)

where C_program_ is the mean percentage control in the programs where fungicide was applied based on the dollar spot warning system presented previously and C_calendar_ is the percentage control in the calendar-based application of fungicide. The within-study variance (*S*_*i*_^*2*^) was also calculated by the following equation:

$$S_{i}^{2}=\frac{2\;x\;\text{MSE}}{\text{r}}$$ (5)

where *MSE* is the mean square error, or the residual variance from the ANOVA, and r is the number of replicates in each study. Random-effects (study as a random effect) univariate, meta-analysis was then conducted for each individual model. After initial analysis, subsequent random-effects, univariate, meta-analyses were then conducted for each model with spray action threshold as a moderator. For all meta-analyses (four total analyses) conducted, weight was assigned using the inverse of the within-study variance (1/ *S*_*i*_^*2*^) in the weight statement in PROC MIXED. Due to some cases where programs were evaluated in less than 30 studies (K<30) restricted maximum likelihood method (REML) was used for all analyses [22]. Linear contrast statements were used to obtain mean effect sizes, standard errors (SE), and 95% confidence intervals. Finally, a *t*-statistic was calculated to determine if $\bar{C}$ was different from zero.

## Results

### Dollar spot warning system development

From 2008 through 2010 there were 7,182 observations collected for use in model development (Table 1). Though only the meta-analysis results are presented here, all the data collected from all the sites used to develop the model can be accessed at the Dryad data depository (doi:10.5061/dryad.9m771). After observations were combined across replicates for each rating at each site, 1,184 observations were used to develop the most effective predictive models. Variables most associated with disease included the binomial fungicide application variable (FUNG), mean daily air temperature (AT), mean daily relative humidity (RH), maximum daily relative humidity (MAXRH), minimum daily soil temperature (MINST), and minimum daily air temperature (MINAT). Initial model development resulted in a model that included FUNG, RH, AT, MINST, and MINAT. However, multi-collinearity was identified between some variables in the initial model and MINST and MINAT were subsequently removed from the analysis and a new model was developed using FUNG, RH, and AT (Table 3). Deviance residual plots indicated that two data points out of 1,184 were out of range with the rest of the data. These points were investigated and found to be incorrect temperature readings at the OJ Noer site on 29 July 2009 due to a hardware failure. These two points were removed from the data set and subsequent residual plots were considered satisfactory. Reduced models were also investigated using FUNG, plus either RH or AT and found to be comparable to the full model in describing dollar spot events (Table 3). The highest maximum rescaled R^2^ value and area under the ROC resulted from the full model, containing FUNG, RH and AT, indicating that this model might be the best to implement in the field. Additionally, Wilson (2011) found that *S*. *homoeocarpa* was able to grow across a wide range of temperatures under controlled conditions, indicating that the AT model might not be as accurate in predicting dollar spot epidemics [32]. Relative humidity was shown to strongly influence *S*. *homoeocarpa* growth in controlled conditions as the fungus had limited growth below 70% RH [33]. Thus, the RH model and ATRH model were investigated further in model validation experiments. ROC analysis to determine potential action thresholds for making fungicide recommendations using the RH and ATRH models was investigated according to Hosmer and Lemeshow [27]. Initial action thresholds were set at 30% for the RH model and 20% for the ATRH model.

**Table 3:** Best fitting models describing the probability of a dollar spot event using the binomial fungicide application variable (FUNG; where 1 = fungicide applied and 0 = no fungicide applied), mean daily air temperature (AT), and mean daily relative humidity (RH) as independent variables^z^. Disease and weather data for model development was collected from the OJ Noer Turfgrass Research Facility in Madison, WI and the Oklahoma State University Turfgrass Research Facility in Stillwater, OK between 2008 and 2010.

Model^y^	Max-rescaled R^2^	ROC^x^
logit (μ) = -8.139–1.079 FUNG + 0.099 RH	0.19	0.74
logit (μ) = -4.944–1.155 FUNG + 0.213 AT	0.19	0.73
logit (μ) = -11.404–1.139 FUNG + 0.089 RH + 0.193 AT	0.26	0.77

^z^ Models were developed using the STEPWISE procedure in SAS (SAS Institute, Cary, NC).

^y^ Data used were collected during the 2008 through 2010 field seasons; n = 1182.

^x^ ROC = Area under the receiver operator curve, where a higher ROC indicates good sensitivity and specificity of the model.

### Validation of the dollar spot warning system in the field

Dollar spot infection center development was highly variable from one location to the next and across each season so a meta-analysis of the ATRH and RH models was conducted for all field sites between 2010 and 2016 to determine the most effective predictive model (Table 2). A total of 26 site-years of data were used to validate the dollar spot prediction models used to advise fungicide applications. Neither model provided control similar to a calendar-based program when results from all spray thresholds were combined (Table 4). However, when spray thresholds were taken into account both models provided control similar to that of a calendar-based program with at least one threshold level. The RH model provided similar control only at the 10% threshold (Table 5). The ATRH model provided similar control to the calendar-based approach at the 10, 15, and 20% thresholds (Table 6). The ATRH 20% threshold performed best out of all model/threshold combinations and was chosen as the standard for additional field validation research. When just the ATRH 20% threshold was compared to a calendar-based program (K = 34) over multiple sites and multiple years, the dollar spot warning system provided control of dollar spot foci similar to the calendar-based approach (Table 7).

**Table 4:** Analysis of 89 (k = 89) replicated studies comparing dollar spot control using a logistic model-based approach to schedule fungicide applications with a calendar-based approach without accounting for spray decision threshold. Different environmental variables included in the logistic model-based approach were a combination of mean daily air temperature and relative humidity (ATRH) and mean daily relative humidity (RH).

Model	^$\bar{C}$^^z^	SE	t-value	*P*-value^y^	95% Confidence Interval	
					Lower	Upper
RH	-16.24	7.39	-2.2	0.028	-30.72	-1.76
ATRH	-17.36	3.74	-4.64	<0.001	-24.68	-10.03

^z^ Mean dollar spot control effect size for each model relative to the calendar-based approach. More negative numbers indicate lower control in the model treatment relative to the calendar-based program.

^y^ *P*-value ≥ 0.05 indicates that the number of dollar spot foci in the model treatment do not differ from the number in the calendar-based approach.

**Table 5:** Analysis of 16 (k = 16) replicated studies comparing dollar spot control using the mean daily relative humidity (RH) logistic dollar spot model to a calendar-based approach at spray threshold probabilities of 10, 20, and 30%.

Threshold (%)	$\bar{C}$^z^	SE	t value	*P*-value^y^	95% Confidence Interval	
					Lower	Upper
10	0.003	11.05	0.00	0.999	-21.66	21.67
20	-19.16	7.49	-2.56	0.011	-33.85	-4.48
30	-27.95	13.66	-2.05	0.041	-54.73	-1.16

^z^ Mean dollar spot control effect size for each model relative to the calendar-based approach. More negative numbers indicate lower control in the model treatment relative to the calendar-based program.

^y^ *P*-value ≥ 0.05 indicates that the number of dollar spot foci in the threshold treatment do not differ from the number in the calendar-based approach.

**Table 6:** Analysis of 62 (k = 62) replicated studies comparing dollar spot control using the mean daily air temperature and mean daily relative humidity (ATRH) logistic dollar spot model to a calendar-based approach at spray threshold probabilities of 10, 15, 20, 25, 30, 40, 50, and 75%.

Threshold (%)	$\bar{C}$^z^	SE	t value	*P*-value^y^	95% Confidence Interval	
					Lower	Upper
10	-11.26	9.39	-1.20	0.230	-29.66	7.14
15	-6.86	18.53	-0.37	0.711	-43.18	29.46
20	-5.91	4.08	-1.45	0.147	-13.90	2.08
25	-40.64	18.53	-2.19	0.028	-76.96	-4.32
30	-29.57	6.76	-4.38	<0.001	-42.82	-16.33
40	-103.8	25.86	-4.01	<0.001	-154.50	-53.10
50	-101.6	25.86	-3.93	<0.001	-152.30	-50.90
75	-85.9	25.86	-3.32	<0.001	-136.60	-35.20

^z^ Mean dollar spot control effect size for each model relative to the calendar-based approach. More negative numbers indicate lower control in the model treatment relative to the calendar-based program.

^y^ *P*-value ≥ 0.05 indicates that the number of dollar spot foci in the threshold treatment do not differ from the number in the calendar-based approach.

**Table 7:** Dollar spot foci effect size in 34 (k = 34) replicated trials where fungicide was applied based on the ATRH model using a 20% threshold compared to the calendar-based approach.

$\bar{C}$^z^	SE	t value	*P*-value^y^	95% Confidence Interval	
				Lower	Upper
-4.61	3.25	-1.42	0.156	-36.31	27.74

^z^ Mean dollar spot control effect size for each model relative to the calendar-based approach. More negative numbers indicate lower control in the model treatment relative to the calendar-based program.

^y^ *P*-value ≥ 0.05 indicates that the number of dollar spot foci in the model treatment do not differ from the number in the calendar-based approach.

**Fig 1:**
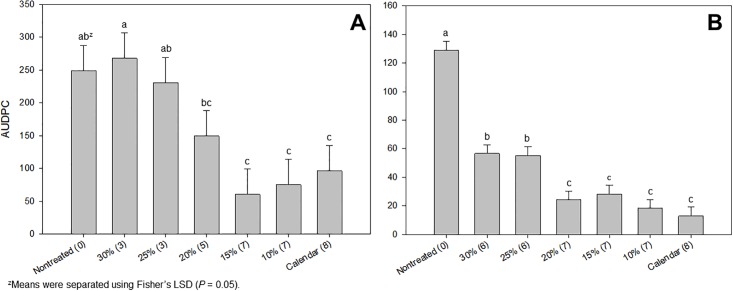
Dollar spot infection centers as assessed by area under the disease progress curve (AUDPC) in 2015 (A) and 2016 (B) in response to various logistic model treatment thresholds at the OJ Noer Turfgrass Research and Education Facility in Madison, WI. All treatments except for the non-treated consisted of propiconazole applied at the rate of 1.00 kg a.i. ha^-1^ and were assumed to have a 21-day duration of efficacy. Number of applications made per treatment are in parentheses following the treatment name on the x-axis. Error bars represent the standard error about the mean.

The ATRH dollar spot warning system provided effective dollar spot control while requiring fewer fungicide applications in both years of a 2-year threshold refinement study at the OJ Noer Turfgrass Research Facility (Fig 1). In 2016 the 20% threshold was the highest threshold that still provided dollar spot control statistically similar to the calendar-based program while in 2015 the 20% threshold was statistically similar to the calendar-based program and the non-treated control. In 2015, the ATRH dollar spot warning system using the 20% threshold resulted in the savings of three fungicide applications compared to the calendar-based program, while the same warning system in 2016 resulted in the savings of one fungicide application compared to the calendar-based program (Fig 1).

## Discussion

The results provided here demonstrate that a logistic-based dollar spot warning system, requiring only the measurement of average daily air temperature and average daily relative humidity, can accurately advise fungicide program applications that provide control of dollar spot comparable to that of a calendar-based program. In addition, the model also facilitates the use of fewer fungicide applications compared to a calendar-based approach. The fungicide Banner MAXX (active ingredient of propiconazole) retails for approximately $356 ha^-1^ USD ($144 acre^-1^ USD), and a typical golf course in the U.S. has approximately 12.1 ha (30 acres) of fairways [34]. So even the savings of a single application will save the superintendent $4,300 USD on their fairway pesticide program, and the 3-application reduction compared to the calendar-based approach observed in the 2015 threshold refinement study would save the golf course nearly $13,000 USD. This savings only accounts for the cost of product, and additional savings would likely be obtained from lower fuel usage and labor savings, not to mention the reduced environmental impact of decreasing pesticide use.

The greatest fungicide savings using the dollar spot warning system will likely be observed in locations that do not experience constant favorable conditions for dollar spot development. In many temperate locations the savings may be during the transitional growing periods of spring and fall when temperatures and relative humidity are lower. For example, in the 2015 threshold refinement study, the calendar-based program scheduled three fungicide applications after September 1^st^ but the dollar spot warning system using the ATRH model at a 20% action threshold only advised one fungicide application in that same period. On the contrary, in 2016 the calendar-based method scheduled three fungicide applications after September 1^st^ but the dollar spot warning system using the ATRH model at a 20% action threshold also advised three fungicide applications, leading to no fall fungicide savings. This lack of savings was the result of a warmer and more humid summer and extended fall weather in 2016 (mean Aug through Oct temp/dew point of 17.8°C/13.3°) compared to the relatively mild summer of 2015 and seasonable fall (mean Aug through Oct temp/dew point of 17.2°C/11.1°). Savings may also be obtained during the summer months in areas that typically experience hot summer conditions that are sub-optimal for *S*. *homoeocarpa* growth. Temperatures consistently in excess of 30°C will limit dollar spot development, and the warning system can be used to determine when conditions are too warm for dollar spot to occur [3]. Thus, in locations or years where the conditions are consistently favorable for dollar spot to occur the dollar spot warning system will not result in significant fungicide spray savings over the calendar-based program. However, in locations or years where environmental conditions become unfavorable for infection there can be great financial and environmental benefits in using the predictive model to schedule dollar spot fungicide applications. In short, this dollar spot warning system helps golf course superintendents apply fungicides for dollar spot control only when they are needed in a wide range of environments.

In addition to the development of an effective disease prediction model, an accurate and efficient means to collect weather data is also required before a disease warning system can be widely incorporated. The Integrated Plant Protection Center at Oregon State University manages USPest.org, which supplies nationwide weather information and pest and disease models for over 6,300 weather stations. The ATRH disease warning system reported here functions nationally through this portal (http://uspest.org/risk/models) and provides an automated dollar spot prediction for a specific location. This site, and others like it, may become a valuable asset for turf managers around the United States for more accurately timing fungicide applications to dollar spot symptoms development.

Many current disease warning systems use a qualitative tool to inform growers when a fungicide should be applied [13, 25]. The disease warning system presented in this paper provides a fully quantitative assessment of the probability of dollar spot development, and our research suggests that 20% is the most effective initial action threshold to use in scheduling fungicide applications. In practice, however, the most effective threshold will likely vary across locations. Most fungal plant pathogens cause more disease in areas of limited air movement due to prolonged periods of leaf wetness [35]. Locations on a golf course where a weather station is in a relatively open area with ample air movement will likely under predict disease on areas of the facility where air movement is restricted or drainage is poor, resulting in potentially poor dollar spot control using the 20% threshold and ATRH warning system. The opposite would likely be true if the weather station is placed in an area of high disease pressure, and more fungicide may be used across the golf course than is necessary to suppress disease. Additional cultural variables such as host species, cultivar resistance, fertility level, soil type, irrigation practices, and numerous others will all undoubtedly impact the development of dollar spot and the application threshold that works well for that particular course. In this sense, the use of a quantitative model allows for each user to determine the precise threshold that provides an acceptable level of disease control for their particular location. This approach requires a larger upfront time investment by the end user to determine their particular threshold, but a greater likelihood of future savings through more targeted fungicide applications.

Financial, social, and regulatory pressure to reduce the reliance on chemical disease control will likely continue to increase for golf course superintendents and other plant managers in the years to come. Development of logistic-based disease warning systems has been successful in other agronomic and horticultural systems, and it can be successful for management of dollar spot in golf course systems as well. Further research into the ecology and epidemiology of the dollar spot pathogen is warranted to refine the predictive model presented here and to potentially identify areas for alternative (i.e. non-chemical) disease control. In addition, for this and other disease warning systems to truly become an integral tool for golf course superintendents there must be advances in weather monitoring technology and software that allow for site-specific analysis of disease activity in a user-friendly manner. Many of these advances are already occurring in the agricultural industry, and their continued adoption by the turfgrass industry will make widespread adoption of weather-based disease control approaches attainable in the near future.
